# Identification of the skin microbiome as an emerging and modifiable risk factor for radiation dermatitis in breast cancer

**DOI:** 10.1007/s00520-024-08747-1

**Published:** 2024-07-23

**Authors:** Cas Stefaan Dejonckheere, Julian Philipp Layer, Leonard Christopher Schmeel, Eleni Gkika

**Affiliations:** 1 Department of Radiation Oncology, University Hospital Bonn, Venusberg-Campus 1, 53127 Bonn, Germany; 2 Institute of Experimental Oncology, University Hospital Bonn, 53127 Bonn, Germany

Acute radiation dermatitis (RD) is the most common side effect of radiotherapy for breast cancer, affecting the majority of patients [1]. Ionizing radiation disrupts the self-renewing properties of the epidermis, thus impairing its barrier function and making patients more susceptible to infection. The clinical appearance is characterized by erythema, swelling, pruritus, discomfort, and dry or moist desquamation [2]. Severe cases with erosions and ulcerations might even require interruption or premature termination of radiation treatment, hampering tumour control outcomes. Following ongoing advancements in radiation treatment technique, dose fractionation, patient positioning, and de-escalation resulting from improved understanding of recurrence patterns, the latter has become rare; however, even mild symptoms are known to majorly impact quality of life and self-image of breast cancer patients [3]. Several trials have investigated patient-specific predictive factors associated with RD, identifying body mass index, breast volume, smoking habits, and diabetes mellitus [4–6]. The majority of these are non-modifiable risk factors, which do not allow any specific starting point for preventative or therapeutic approaches, which are clearly needed in this context [7].

Two recently published brief reports identified the skin microbiome as a new and potentially modifiable risk factor for RD in patients undergoing radiotherapy for breast cancer. In an initial report, Kost et al. showed that nasal *Staphylococcus aureus* colonization may be associated with RD severity [8]. Furthermore, Hülpüsch et al. demonstrated that in patients developing severe RD, an overgrowth of non-species-specific skin bacteria was observed before the onset of severe symptoms [9]. The exact interplay between the radiation-damaged skin barrier and the skin microbiome (both its absolute load as well as its composition) is not yet fully understood and might go beyond a mere increased susceptibility for local infections. Both trials, however, suggest the further investigation of the skin microbiome composition as a promising path towards a long-sought potential predictive biomarker. Specific cancer patient predispositions (e.g. immune status) are usually non-modifiable, yet these new insights might enable easily applicable personalized prevention strategies.

So far, two trials have investigated preventative strategies based on microbial decolonization. The follow-up trial by Kost et al. tested this rationale and investigated the use of a chlorhexidine body cleanser (applied once daily for five consecutive days prior to radiotherapy and then for five days every two weeks throughout treatment) to achieve bacterial decolonization [10]. This preventative regimen was associated with significantly reduced clinician-reported RD severity, along with the expected significantly lower rates of colonization with *Staphylococcus aureus* in comparison with the standard of care group. A single patient only experienced an adverse event (itching) related to the intervention. Another promising RD prevention strategy based on microbial decolonization is the topical application of non-invasive physical plasma (NIPP). This very well-tolerated partially ionized gas characterized by free electrons and generated out of ambient air has shown promising results in a phase 1 and 2 trial, with a significant positive impact on both patient- and clinician-reported outcomes in breast cancer patients [11, 12]. NIPP is already routinely applied for the treatment of chronic wounds such as diabetic foot ulcers, where a significantly improved wound healing was denoted across multiple phase 3 trials, partly owing to the significant reduction of the bacterial load compared to baseline assessments [13, 14]. Furthermore, NIPP induces the proliferation and migration of keratinocytes, fibroblasts, and endothelial cells, through the expression of wound healing and tissue regenerating gene signatures. Contrary to the above-mentioned chlorhexidine, NIPP has no known side effects, making it a very appealing prevention method. A randomized double-blind placebo-controlled phase 3 trial of NIPP in breast cancer patients undergoing adjuvant radiotherapy is currently ongoing and will unequivocally establish its role in RD prevention (NIPP-RD III; ARO-2024–07; DRKS00032560).

The impact of certain shifts in the composition of the skin microbiome following radiotherapy is also of interest. A reduced microbial diversity leads to an overrepresentation of certain taxa, the metabolic abilities of which have been shown to modify the RD risk (e.g. resulting in delayed recovery or higher tendency towards developing chronic RD) [9, 15, 16]. The impact of the above-mentioned preventative strategies on the microbial composition as well as new potential approaches is worthy of further investigation. Future trials will hopefully further elucidate the interplay between host and skin microbiome in the complex pathogenesis of RD and add to the emerging evidence of the skin microbiome as a modifiable risk factor for radiation dermatitis in breast cancer.

## Data Availability

No datasets were generated or analyzed during the current study.
